# Hepatitis B virus DNA polymerase gene polymorphism based prediction of genotypes in chronic HBV patients from Western India

**DOI:** 10.4314/ahs.v17i3.19

**Published:** 2017-09

**Authors:** Yashwant G Chavan, Sharad R Pawar, Minal Wani, Amol D Raut, Rabindra N Misra

**Affiliations:** 1 Dr. D Y Patil Biotechnology and Bioinformatics Institute, Tathwade, Pune 411033. Dr. D Y Patil Vidyapeeth, Sant Tukaram Nagar, Pimpri, Pune 411018. Maharashtra, India; 2 Medical Genetics, geneOmbio Technologies Private Limited, Baner, Pune 411045. Maharashtra, India; 3 Department of Microbiology, Dr. D Y Patil Medical College, Dr. D Y Patil Vidyapeeth, Sant Tukaram Nagar, Pimpri, Pune 411018. Maharashtra, India

**Keywords:** Hepatitis B virus, nested PCR, genotype, sub-genotypes, YMDD mutations

## Abstract

**Background:**

Hepatitis B Virus (HBV) infection is one of the major causes of liver cirrhosis, hepatocellular carcinoma and deaths due to the acute or chronic consequences worldwide. HBV is distributed into various genotypes based on nucleic acid sequence variation.

**Objectives:**

To develop a method of HBV genotyping and drug resistance interpretation using partial sequencing of polymerase gene.

**Methods:**

This study was performed on 98 HBV infected patients' serum samples from Western India. A nested PCR protocol was designed for amplification of pol gene from HBV genome and Sanger's sequencing of the gene fragment. Sequences were aligned with HBV reference sequences for phylogenetic analysis and for characterization of genetic diversity. Drug resistance mutations were screened using HBVSeq program from Stanford University.

**Results:**

Distribution of HBV genotypes showed predominance of genotype D, circulating in 76 (77.55%) patients (p < 0.05). Genotypes A and C were less prevalent and were identified in 4 (4.08%) and 18 (18.37%) patients, respectively. Anti-retroviral drug resistance mutations were not detected in any patient.

**Conclusion:**

A method for determination of HBV genotypes using pol gene sequencing which simultaneously detects major drug resistance mutations has been established. HBV genetic diversity may play an important role in treatment decision.

## Introduction

HBV infection is a major health care problem with up to 400 million affected persons worldwide and accounts for one million deaths worldwide from cirrhosis, liver failure and hepatocellular carcinoma.^1^ HBV has been broadly classified into eight genotypes, designated as A–H.^2^ This classification is based on inter-genotypic divergence of at least 8% in the complete nucleotide sequence or more than 4% in the S gene.^3^ Evidence from various research studies has suggested that HBV genotypes may play some role in causing different disease profiles in chronic hepatitis B (CHB).^4^ Among Asians, who constitute greater than 75% of the worldwide population of individuals with CHB, genotypes B and C are the two most common HBV genotypes in Asia and Oceania.^5^ Genotype D is most widely distributed and found in Africa, Europe, Mediterranean countries, India, Indonesia and Nigeria.^6^

Correlation of genotypes with severity of infection has also been established. Researchers have found the association of genotype C with more severe liver disease as compared to genotype B and higher incidence of HCC in genotype D infected patients.^7^ In Europe, most patients with genotype D are reported to have acute hepatitis B, while most patients with genotype A have chronic hepatitis B.^8^ Association study in patients on Lamivudine therapy showed that patients with genotype D achieved higher SVR (Sustained viral response) than with genotype A; hence response to therapy is also found to be ruled by genotypes.^9^ Due to these important findings, HBV genotyping has gained immense importance in guiding treatment decisions, improving vaccination, and controlling liver diseases. Various methods of genotyping are available of which sequencing of S gene was mostly performed in the previous studies. Ying et al., performed semi-nested PCR amplification (using three primers) of pol gene and its sequencing for HBV genotyping in Chinese patients.^10^ In this study, we used nested PCR that uses four primers that results in increased specificity. As this assay is performed on low viral load samples, it also underlines higher sensitivity of nested PCR approach. Advances in molecular biology techniques have led to implementation of real time PCR for detection of HBV genotypes using melting curve analysis.^11^ This study comprises HBV polymerase gene sequencing covering YMDD region which also includes the overlapping S gene region, it was found that the sequence can be used to identify the genotype of the virus. This genotyping method makes use of Sanger's DNA sequencing technology to sequence HBV DNA polymerase region using different set of primers to amplify the HBV DNA polymerase and overlapping S gene region. Along with genotyping of Hepatitis B virus, this method also helps in determination of drug resistance mutation in YMDD hot-spot region of DNA polymerase gene.

## Materials and methods

### Study participants

Four hundred and twenty seven chronic HBV infected patient serum samples were received at Molecular Diagnostic Lab of geneOmbio Technologies Private Limited (Pune, India) during March 2012 to May 2014 from associated hospital laboratories located in Western Indian states of Maharashtra, Goa and Gujrat. This was a retrospective cross-sectional study and all procedures conformed to the ethical guidelines of the 1975 Helsinki Declaration. No personal identification information or other personal identifiers such as address or hospital identification number were recorded to ensure patient confidentiality. This study was approved ethically by the Institutional Review Board (IRB) of geneOmbio Technologies (Approval No. IRB/2012/G-011) and Institutional Biosafety Committee (IBSC) at Dr. D. Y. Patil Vidyapeeth, Pune. The serum samples were collected and stored at −40°C until use.

### Inclusion criteria

Patients with HBeAg and HBsAg positive sera confirmed by HbeAg/HbsAg Rapid Test Kit (Medical Biological Services, Italy) and having viral load in the range of 7-110000000 HBV DNA IU/mL were included. HBV viral load was determined using Roche COBAS TaqMan 48 Kit on COBAS TaqMan 48 Analyzer (Roche Molecular Systems, USA).

### Exclusion criteria

Patients positive for Hepatitis C virus (HCV) and Human Immunodeficiency virus (HIV) anti-body test were excluded from the study. Screening for HCV and HIV-1 was done using commercially available HCV TRI DOT and HIV TRI DOT kit (J. MITRA &Co. Ltd., New Delhi, India). Patients having viral load undetectable or less than 7 HBV DNA IU/mL were also excluded from the study. Of the 427 samples, 140 patients were both HBeAg and HBsAg positive. Thirty eight of these 140 patients had HBV viral load undetectable or less than 7 HBV DNA IU/mL, therefore were excluded from the study. Four samples were excluded due to co-infection with either HCV or HIV.

Hence this retrospective cross-sectional study examined only a total of 98 (70 males, 28 females, mean age 43.8 + 11.6 years) patients.

### Primer design

Twenty three full-length HBV sequences representing all of the available HBV genotypes were downloaded from GenBank nucleotide sequence database (http://www.ncbi.nlm.nih.gov). These sequences were selected from list provided by NCBI Genotyping tool (http://www.ncbi.nlm.nih.gov/projects/genotyping) as reference set of sequences for HBV genotyping. Using ClustalW multiple sequence alignment tool the reference sequences were aligned to find the conserved domains.^12^ First round PCR primers YMDDF1 (5′- CAAGGTATGTTGCCCGTTTG-3′) and YMDDR1 (5′-CCCAACTCCTCCCAGTCCTTAA-3′) were selected from previously published data.^13^,^14^ With the help of Primer 3- Online Primer designing tool (http://primer3.ut.ee), we designed a nested PCR primer pair YMDDF2 (5′ -CTGTATTCCCATCCCATCATC-3′) and YMDDR2 (5′-GACCCACAATTCGTTGACATAC-3′). The primers were synthesized at Eurofins Genomics Pvt. Ltd. (Bangalore, India). The amplified fragment of the polymerase gene overlaps slightly with the S gene. First round PCR primers were expected to generate 1290 bp amplicons whereas the nested PCR primers were expected to generate 409 bp amplicons. Implementation of nested PCR protocol enhances the sensitivity of the assay as it can amplify samples having very low HBV DNA viral load.

### DNA isolation, PCR and sequencing of HBV polymerase gene

DNA isolation from serum samples was performed using High Pure Viral Nucleic Acid Kit (Roche Molecular Systems, USA) according to the manufacturer's instructions. PCR amplification was performed using Platinum Taq DNA Polymerase (Invitrogen Corporation, USA).

The fragment of the polymerase gene was amplified by nested PCR with two rounds of amplification. Five mi croliter of DNA isolated from each patient was added to a 20 µL PCR mixture. The PCR mix contained 2.5 µl of 10x PCR buffer (100 mM Tris- pH 9.0, 500 mM KCl, 15mM MgC_l2_ and 0.1% Gelatin), 200 µM dNTPs, 1 unit of Platinum Taq DNA polymerase, 20 pM each of YMDDF1 and YMDDR1 primers for first round PCR and sterile nuclease free water to make a final volume of 25 µl. The first round of amplification was performed with an initial 5 min denaturing step at 95°C, followed by 30 cycles of denaturing for 45 s at 94°C, annealing for 30 s at 60°C, and elongation for 1 min at 72°C, with a final extension period of 10 min at 72°C. The second round of amplification was performed using 20 pM each of YMDDF2 and YMDDR2 primers, with an initial 5 min denaturing step at 94°C, followed by 30 cycles of denaturing for 45 s at 94°C, annealing for 30 s at 55°C, and elongation for 30 s at 72°C, with a final extension period of 10 min at 72°C. The reaction products of the nested PCR were visualized on a 2% (w/v) agarose gel stained with ethidium bromide. Purification of nested PCR products was performed using Purelink PCR product purification kit (Life technologies, USA). Automated DNA sequencing was performed using BigDye Terminator v3.1 Cycle sequencing kit on 3130 Genetic Analyzer (Applied Biosystems, USA). Nucleotide sequences were submitted to NCBI nucleotide sequence database GenBank.

### Determination of HBV Genotypes and phylogenetic analysis

NCBI HBV genotyping tool (http://www.ncbi.nlm.nih.gov/projects/genotyping) was used for determination of genotype for each HBV sequence. Nucleotide sequences of 336 bases generated after sequence quality trimming using Chromas Pro 1.34 (Technelysium Pty. Ltd., Queensland, Australia) were used for homology and phylogenetic analysis. Sequences were aligned by using the ClustalW program. Two representative reference sequences from each genotype were used for construction of phylogenetic tree (A: AF418677, X51970; B: D23678, AB073838; C: D50489, Y18855; D: AB090270, AF151735; E: AB032431, X75657; F: AB036905, AB036907; G: AB064313, AB056513 and H: AY090454, AY090457). The alignment was converted in MEGA format using MEGA 6.06 software.^15^ The phylogenetic tree was constructed by the Maximum composite likelihood algorithm with a bootstrap test of 1000 replicates.^16^ Significance of polymerase gene fragment sequence for genotyping was evaluated by phylogenetic analysis of reference sequences of all genotypes (A-H) obtained from NCBI genotyping tool reference set and GenBank database. This reference set includes total 66 sequences.

### Genetic distance

An analysis of the number of base substitutions per site between the nucleotide sequences (genetic distance (d)) was conducted using the maximum composite likelihood method in MEGA 6.06 for reference sequences from each genotype used for phylogenetic analysis. Analyses were conducted using the Maximum Composite Likelihood model. The differences in the composition bias among sequences were considered in evolutionary comparisons. The analysis involved 16 nucleotide sequences. Similarity matrix was derived from the distance analysis.

### Evolutionary substitution rates

Different genotypes are evolved due to substitution mutations at different sites within the viral genome.^17^ To determine the sites that are highly prone to substitutions, we performed the substitution rate analysis using MEGA 6.06. Substitution pattern and rates were estimated under the Tamura-Nei (1993) model (+G).^18^ A discrete Gamma distribution was used to model evolutionary rate differences among sites (5 categories, [+G]).^19^

### Determination of drug resistance mutations

Drug resistance interpretation based on mutation in HBV polymerase gene was inferred by submitting the sequence data to Stanford University database (http://hivdb.stanford.edu/HBV/HBVseq) and Geno2pheno (hbv) resistance prediction tool (http://hbv.geno2pheno.org) (Max Planck Institute)

### Statistical analysis

Clinical characteristics, mean and frequency of genotyping data were analysed using a SPSS statistics (2014) and Microsoft Excel (2013). Descriptive statistics are presented as proportions, means ± standard deviation, and medians with interquartile range. Comparative analyses were performed using the Student's t-test and Chi square test. A P value of less than 0.05 was considered significant.

## Results

Clinical characteristics of study patients infected with HBV are provided in Table 1. All the patients were from state of Maharashtra geographically located in Western India. Mean HBV viral load of study samples was 4.46 + 1.77 HBV DNA IU/mL.

**Table 1:** Clinical characteristics of study patients infected with Hepatitis B Virus

Variables	Population (n= 98)
**Age, years**	43.8 ± 11.6
**Male/Female, n (%)**	70/28 (71.43%/28.57%)
**HBsAg, positive**	98 (100%)
**HBeAg, positive**	98 (100%)
**Serum ALT (U/L) ± SD**	86.10 ± 32.54
**HBV DNA positive, n (%)**	98 (100%)
**HBV viral load, log_10_ IU/mL** **(range, HBV DNA IU/mL)**	4.46 ± 1.77 (7-110000000 HBV DNA IU/mL)

## PCR amplification of HBV pol gene

**Figure 1:**
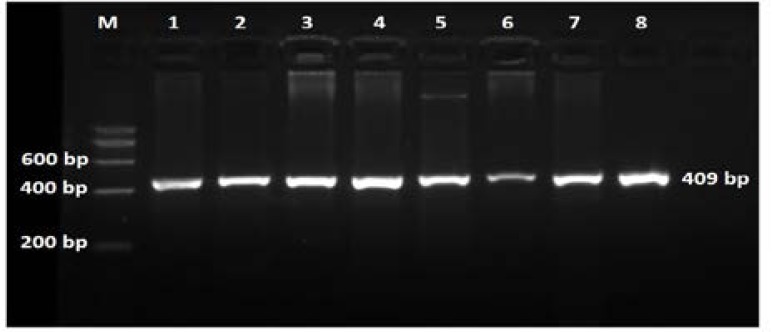
Agarose gel electrophoresis (2% w/v) of the nested PCR products and DNA marker. 200 bp DNA ladder (Lane M), second round of amplification products (Lane 1–8).

Hepatitis B Virus DNA isolated from patients generated 409 bp PCR amplification product for pol gene using nested PCR approach (Figure 1). In total, all 98 (100%) samples were positive for polymerase gene fragments. Each of these nested PCR reaction products were then sequenced. The nucleotide sequence data was submitted to GenBank, these sequences are available at NCBI nucleotide sequence database with accession number KM076939-KM077016.

## Determination of HBV genotypes and phylogenetic analysis

Three major genotypes A, C and D were found within the study population as predicted by NCBI genotyping tool for HBV. HBV Genotype D (n=76; 77.55%) was found to be predominant circulating genotype (p < 0.05), followed by C (n=18; 18.37%) and A (n=4; 4.08%) among 98 sequences. Geno2pheno and HBVSeq Programs also determined genotypes and subgenotypes of the sequences. Six sub-genotypes were detected A1, C1, C2, D2, D3 and D4, of which HBV sub-genotypes D2 (n=65; 66.33%). Clinical and demographic characteristics of the study population with respect to sub-genotypes distribution are provided in Table 2. Higher levels of Serum ALT (110.33 +42.54 U/L) were observed in sub-genotypes A1. Genotype prediction from NCBI genotyping tool, Geno2Pheno and HBVSeq produced 100% similar results.

**Table 2:** Clinical and demographic characteristic of the study population with respect to sub-genotypes distribution.

Genotype	A (*n=4*)	C (*n=18*)		D (*n=76*)		
Subgenotype	A1	C1	C2	D2	D3	D4
**Number of samples (%)**	4 (4.08)	7 (7.14)	11 (11.22)	65 (66.33)	1 (1.02)	10(10.20)
**Male/Female (%)**	4/0 (100/0)	6/1 (85.71/14.29)	6/5 (54.55/45.45)	45/20 (69.23/30.77)	1/0 (100/0)	8/2 (80/20)
**Age, years (range)**	38.0 ± 11.69	41.57 ± 5.32	43.36 ± 8.49	45.12 ± 12.65	49.0 ± 0.00	39.3 ± 10.88
**Serum ALT (U/L)** **(range)**	110.33 ± 42.54	96.52 ± 40.20	86.00 ± 30.51	82.57 ± 31.96	72.00 ± 0.00	93.57 ± 30.31
**HBV viral load,** **log_10_ copies/mL (range)**	4.17 ± 1.44	4.70 ± 2.03	3.22 ± 1.27	4.51 ± 1.77	3.25 ± 0.00	5.55 ± 1.71
**Common Mutations** **(%)**	I253V (100)	N226H (85), Q267L (85)	S223A (100), I224V(100), V253I (91), Q267H(45), I282V (45)	N248H (49), D263E (23)	N248H (100), C256S (100)	N248H (40), E271D (60)

## Phylogenetic analysis

**Figure 2:**
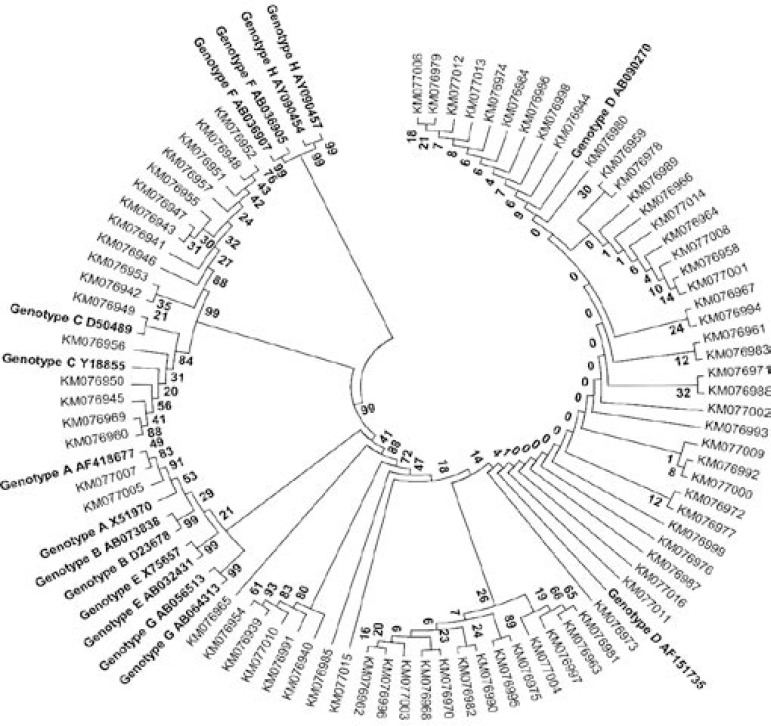
Phylogenetic analysis of Hepatitis B Virus (HBV) based on partial sequence of polymerase gene from 78 study samples and 16 reference sequences representing each genotype. Numbers at respective nodes represent percentage bootstrap values (1000 replicates). The Maximum-Likelihood tree was constructed with a substitution model of Tamura-Nei plus gamma distribution using MEGA software 6.06. As the branch lengths and bootstrap values between various genotype D sequences were low or zero, the tree has been represented in topology view to make ancestor-descendant relationship clear, hence the scale bar was removed.

Phylogenetic analysis based on alignment of nucleotide sequences of HBV polymerase gene from 78 study samples and 16 representative reference sequences (NCBI GenBank database) was used to confirm the presence of genotype A, C and D. As evident from phylogenetic tree presented in Figure 2, Genotype A, C and D samples clustered with closest reference sequences of the same genotype. The evolutionary history was inferred using the UPGMA method. Phylogenetic analysis of 66 reference sequences obtained from NCBI GenBank database showed distinct clustering of each genotype proving the significance of use of polymerase gene fragment sequence in HBV genotyping.

**Figure 3:**
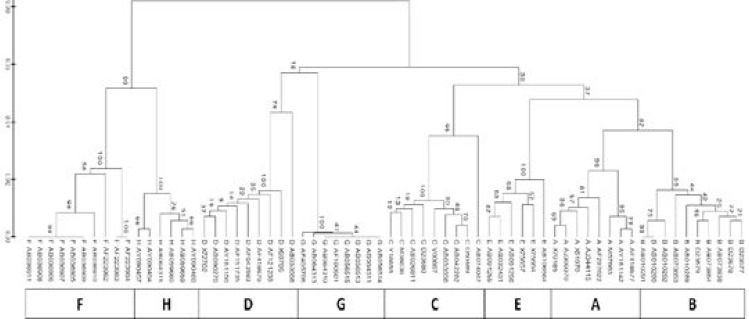
Phylogenetic analysis of 66 reference sequences for polymerase gene obtained from NCBI GenBank database using Maximum-Likelihood Method. Branch reliability is indicated by the percentage of bootstrap values at each node (1,000 replications). The scale bar indicates the number of base substitutions per site. Lower rectangle represent each genotype distributed amongst branches within the tree.

Figure 3 shows distinct branches for each genotype supported by significant bootstrap values.

## Genetic distances

When all eight genotypes were compared to each other (Table 3), HBV genotypes showed genetic divergence ranging from 6.7 (genotype A B) to 18.4 (genotype C → H) based on sequence polymorphism in polymerase gene. The nucleotide sequence similarity ranged from 81.6 (genotype C → H) to 93.3 (genotype A → B). This analysis included two sequences for each genotype hence inter-genotypic and intra-genotypic divergence was also determined.

**Table 3:** Nucleotide sequence similarity and divergence of polymerase gene among HBV genotypes reference sequence obtained from NCBIGenBank database

Genotype	Genotype	D		G		A		B		C		E		F		H	
	Accession number	AB090270	AF151735	AB064313	AB056513	AF418677	X51970	D23678	AB073838	D50489	Y18855	AB032431	X75657	AB036907	AB036905	AY090454	AY090457
D	AB090270		97.7	88.9	89.2	90.5	91.5	88.8	88.9	86.9	87.5	90.2	91.4	84.1	84.1	83.2	83.9
	AF151735	2.3		87.9	88.2	89.3	89.6	86.9	86.9	84.5	85.2	89.6	90.2	83.8	83.8	82.6	83.2
G	AB064313	11.1	12.1		99.7	89.3	89.3	89.2	88.6	86.3	86.7	88.6	88.6	81.7	81.7	82.1	82.5
	AB056513	10.8	11.8	0.3		89.6	89.6	89.6	88.9	86.4	87.0	88.3	88.9	82.1	82.1	82.5	82.9
A	AF418677	9.5	10.7	10.7	10.4		96.6	92.7	92.7	90.5	90.2	91.8	93.4	85.5	85.5	85.3	85.6
	X51970	8.5	10.4	10.7	10.4	3.4		93.3	93.3	90.2	89.8	91.2	92.7	85.5	85.5	85.9	86.3
B	D23678	11.2	13.1	10.8	10.4	7.3	6.7		98.3	91.1	90.5	90.2	91.8	83.7	83.7	84.4	84.7
	AB073838	11.1	13.1	11.4	11.1	7.3	6.7	1.7		91.1	90.8	90.2	91.8	83.7	83.7	84.8	85.1
C	D50489	13.1	15.5	13.7	13.6	9.5	9.8	8.9	8.9		97.5	88.9	89.9	82.8	82.8	82.0	82.3
	Y18855	12.5	14.8	13.3	13.0	9.8	10.2	9.5	9.2	2.5		87.6	88.9	83.2	83.2	81.6	82.0
E	AB032431	9.8	10.4	11.4	11.7	8.2	8.8	9.8	9.8	11.1	12.4		97.4	84.1	84.1	82.9	82.9
	X75657	8.6	9.8	11.4	11.1	6.6	7.3	8.2	8.2	10.1	11.1	2.6		84.8	84.8	83.6	84.3
F	AB036907	15.9	16.2	18.3	17.9	14.5	14.5	16.3	16.3	17.2	16.8	15.9	15.2		100.0	89.6	90.2
	AB036905	15.9	16.2	18.3	17.9	14.5	14.5	16.3	16.3	17.2	16.8	15.9	15.2	0.0		89.6	90.2
H	AY090454	16.8	17.4	17.9	17.5	14.7	14.1	15.6	15.2	18.0	18.4	17.1	16.4	10.4	10.4		99.4
	AY090457	16.1	16.8	17.5	17.1	14.4	13.7	15.3	14.9	17.7	18.0	17.1	15.7	9.8	9.8	0.6	

## Evolutionary substitution rates

All sequences were distributed under five different Gamma categories based on the sequence divergence and types of substitutions. Mean evolutionary rates in these categories were 0.00, 0.05, 0.25, 0.90, 3.80 substitutions per site. The nucleotide frequencies were A = 25.03%, T = 34.81%, C = 19.43%, and G = 20.73%. For estimating ML values, a tree topology was automatically computed. The maximum Log likelihood for this computation was −2972.224. The estimated value of the shape parameter for the discrete Gamma Distribution is 0.2961. These nucleotide substitution rates are scaled such that the average evolutionary rate across all sites is 1. This means that sites showing a rate < 1 are evolving slower than average. Nucleotide positions with substitution rate less than 1 (Mean (relative) evolutionary rate <1) were 237/336 whereas the number of sites with >1 substitution rate (Mean (relative) evolutionary rate >1) were 99/336. Hence the sequence studied has 29.46% nucleotide positions which are evolving faster than average.

## HBV drug resistance interpretation

Genotypic resistance mutations against lamivudine (L180M, M204V/I), adefovir (A181T, N236S), and entecavir (I169M, A184T/V, S202I/G, M250V/I/L) were not detected in any patient. Genotypic variants were detected as other mutations that may not be responsible for drug resistance. The mutations with greater than 5% incidence rate in study population have been provided along with their frequency in Table 4. Mutation at 248^th^ amino acid (N248H) of DNA polymerase gene was the most common genotypic variant found in 32.65% (32/98) of population.

**Table 4:** The mutations in HBV polymerase gene of study samples with greater than 5% incidence rate

Mutation Site	Number of patients with mutation (% Rate)
**N248H**	32 (32.65)
**I253V, D263E**	16 (16.33)
**E271D, V253I**	10 (10.20)
**Q267L, I266R**	7 (7.14)
**I282V, N226H, S213T, I266T, S219A**	6 (6.12)
**Q267H, C256G, N238H, D263S**	5 (5.10)

## Discussion

Traditional HBV genotyping methods using restriction fragment length polymorphism (RFLP) have been developed and used extensively for HBV genotypes A to H.^20^ Genotyping methods that use multiplex PCR with type-specific primers have also been reported.^13^ However, most of these newer methods are based on analyzing the S gene. Here we employed genotyping method based on a segment of the HBV DNA polymerase gene.

This study demonstrated that using HBV DNA polymerase gene sequence all the different genotypes show distinct clades in phylogenetic analysis and hence the gene fragment used for analysis is a suitable target for HBV genotyping. As evident from phylogenetic tree, based on the clusters formed by genotypes A–H, the sequences obtained from HBV DNA polymerase gene from patients can be classified under these genotypes.

In this study, the predominant genotype of Hepatitis B was found to be genotype D, and there were no patients with drug resistant HBV. As per previous reports, genotype C is less reported in India, however we found 18 out of 98 cases to be genotype C which indicates substantial increase in infections with genotype C in Indian population.^21^ The variation in relative frequency of HBV genotypes reported in studies from India by various authors have confirmed that the genotype D has been predominant among others.^22^ Therefore, the fragment of HBV DNA polymerase gene as a target for genotyping hepatitis B in India has been proved to be significant. This genotyping method can also be used to predict antiviral therapeutic response among HBV genotypes and the development of drug resistance due to mutations. The fragment covers clinically important YMDD domain of polymerase gene, mutations which are responsible for failure of anti-viral therapy. Hence using the same sequence data, interpretation of drug resistance in HBV is possible. It is a valuable tool for guiding the treatment of lamivudine-resistant HBV in different clinical settings.^23^ Nucleotide sequence analysis of HBV DNA polymerase gene in all patient samples using different genotyping algorithms detected genotypes A, C and D whereas genotypes B, E, F, G and H were not detected.

Of the eight genotypes of HBV, infection with HBV genotype D is the most common in India. Infection with genotype D is associated with more severe liver disease than genotype A.^24^ The major route of HBV transmission in acute hepatitis seem to be sexual intercourse, frequent international travel and contact with people from different ethnic backgrounds, which may contribute to change in the predominance pattern of genotypes in a geographical location.^25^ This study demonstrated that the patients suffering from HBV infection were infected with different genotypes as D, C and A, suggesting no significant change in predominance of HBV genotype in hepatitis B infected cases.

Drug resistant viral strains are evolved due to increased use of anti-viral drugs to treat chronic hepatitis B, such as lamivudine, adefovir, telbivudine, and entecavir.^26^ In absence of anti-viral drugs and drug pressure, most of the drug resistant mutants do not survive and may not remain as fit as the wild type virus in an acutely infected liver.^27^,^28^ In contrast, natural resistance mutations in treatment-naive chronic hepatitis B patients may exist. Mutation substitution rates in polymerase gene as per our study reveal the number of nucleotide position which are more prone to mutations indicating that such changes may drive incidence of drug resistance as well as different genotypes. Our study was limited to genotype D, C and A due to non-availability of samples from other genotypes, larger studies that include samples from patients with all HBV genotypes are required to represent data for all genotypes.

## Conclusion

The study reports Hepatitis B Virus genotyping using DNA polymerase gene fragment sequencing as an effective tool to determine the HBV genotypes as well as drug resistance mutations in HBV infected patients. This study reports high prevalence of genotype D which is in concordance with previous studies. It provides a useful alternative to complete sequencing of the HBV genome and allows the study genetic variation between various genotypes and drug resistance mutations responsible for anti-viral drug failure. Genotyping data of HBV via sequence of DNA polymerase fragment from various geographical population can be generated to validate this method in different populations. One of the studies reported from China demonstrated the significance of HBV DNA polymerase gene sequencing based genotyping and drug resistance interpretation.^10^ Future studies in patients at different stages of infection and therapeutic treatment would help us in understanding the relevance of genotype, resistance mutations and response of the patient to a particular therapy. These studies can be carried out to investigate the clinical, virological and therapeutical response characteristics of HBV genotypes, with clinical data such that the status of HBV infection can be characterized (carrier, immunotolerant, acute and chronic hepatitis, cirrhosis) using a large population size.

## Abbreviations

CHB: Chronic Hepatitis
S: Sustained viral respon
